# Improving Medication Safety in Cancer Services for Ethnic Minority Consumers: Protocol for a Pilot Feasibility and Acceptability Study of a Co-Designed Consumer Engagement Intervention

**DOI:** 10.2196/49902

**Published:** 2023-09-18

**Authors:** Bronwyn Newman, Melvin Chin, Louisa Robinson, Ashfaq Chauhan, Elizabeth Manias, Carlene Wilson, Reema Harrison

**Affiliations:** 1 Australian Institute for Health Innovation Macquarie University Sydney Australia; 2 South Eastern Sydney Local Health District Randwick Australia; 3 Australia School of Nursing and Midwifery Monash University Melbourne Australia; 4 Olivia Newton-John Cancer Wellness and Research Centre Austin Health Heidelberg, Victoria Australia; 5 Melbourne School of Population and Global Health University of Melbourne Parkville, Victoria Australia

**Keywords:** co-design, ethnic minority, health care equity, patient engagement, patient participation, patient safety, medication safety, medication therapy, medication therapy management, patient transfer, consumer, engagement, intervention, ethnic minority

## Abstract

**Background:**

People from ethnic minorities are often exposed to unsafe care contributing to poorer health care outcomes. Medication safety is a high-risk area requiring intervention to improve care outcomes. Using an adapted, experience-based co-design process with cancer service staff and patients from ethnic minorities, a medication communication tool was created: Making it Meaningful (MiM).

**Objective:**

We aim to test whether the MiM tool is feasible and acceptable for use with ethnic minority consumers in cancer services in Australia.

**Methods:**

A single site, controlled before and after this pilot study, will be used. Patients from Mandarin- and Russian-speaking backgrounds are eligible for inclusion. In total, 40 patients from these cultural backgrounds will be recruited and stratified by language to the intervention and control groups, with 20 participants in the intervention and 20 in the control group. Further, 4 health practitioners will be recruited and trained to use the MiM. Clinicians providing care for patients in the intervention will use the MiM during their usual appointment while providing medication communication using standard care processes for the control group. Telephone surveys will be conducted with participants at 3 time points, T1 before the intervention, T2 1 week post intervention, and T3 1 month post intervention, to assess knowledge and self-efficacy in medication management, perceived usability, and acceptability of the MiM. Qualitative interviews with clinicians who have used the MiM will be conducted 1 month postintervention to explore their perceptions of MiM feasibility and acceptability.

**Results:**

Ethical approval for this research has been provided by the South Eastern Sydney Area Health Human Research Ethics Committee (HRECXXX). Bilingual field-workers, 1 Mandarin-speaking and 1 Russian-speaking, are contacting eligible patients to enroll. It is anticipated that recruitment will be completed by October 2023, with data collection completed by December 2023.

**Conclusions:**

Using experience-based co-design, we identified communication about medication, particularly between appointments, as a key issue impacting the safety of care for patients from ethnic minorities accessing cancer services. Increasing consumer engagement in medication management was identified as a strategy to reduce medication safety problems in cancer care; the MiM strategy was developed to address this issue. It is anticipated that by using the MiM, patient knowledge about prescribed medications and confidence in medication management will increase. Evidence from the pilot study will be used to inform a full-scale trial of the MiM tool with a range of ethnic minority communities accessing cancer services. A full-scale trial will seek to determine whether the MiM intervention is effective in knowledge and confidence about medication management, but also whether this improves patient outcomes in cancer care.

**Trial Registration:**

Australian New Zealand Clinical Trials ACTRN12622001260718p; https://www.anzctr.org.au/Trial/Registration/TrialReview.aspx?id=384658&isReview=true

**International Registered Report Identifier (IRRID):**

DERR1-10.2196/49902

## Introduction

### Background

People from ethnic minority backgrounds can experience more unsafe care in health services than the general population. Specifically, current evidence indicates that people from ethnic minority backgrounds experience a higher rate of medication errors, infections, and face systemic barriers to communication with health care providers [1,2]. A range of factors are identified as contributing to experiences of unsafe care. These include; language proficiency, individual or organizational health literacy, socioeconomic factors, and ineffective communication and collaboration between health professionals, patients, and their families [1].

Medication management has been identified as a particular challenge to patients and the family and friends who support them. In this protocol, the family, caregivers, or friends who provide this support will be referred to as carers. More effective engagement between patients and health care providers has been identified as an important strategy to address this issue in several studies [3-5]. For example, medication errors can be reduced by increasing patients’ knowledge about the purpose and use of their medications via supply of tools such as handheld medication lists [6]. Evidence of unsafe care among ethnic minority communities suggests that periods of transition in care (eg, hospital discharge and discharge from emergency department) pose particular risks to patient safety that may be addressed by improved patient engagement [7]. Hospital discharge is a period of high-risk for communication errors relating to medication [3,4]. Although there are strategies currently available to manage patients’ participation in this transition, which are collectively described as patient engagement strategies, the potential for cultural and language diversity to moderate the effectiveness of these strategies is an important issue requiring further research [8,9].

Involving patients and carers in health care decision-making and care planning has been shown to improve health care safety in the general population [10-12]. Common approaches include encouraging patients and family members to raise questions or concerns with health professionals, to engage in learning about how to manage their own health, and to use preformatted tools or resources to plan care or guide decision-making processes [11]. Current patient engagement strategies rely heavily on effective individual communication skills and health literacy among patients and families. A recent review of strategies used to involve patients and families at point-of-care highlighted that none of the 26 identified strategies were explicitly developed for, or evaluated with, people from ethnic minority backgrounds [8]. In further work to explore the appropriateness of patient engagement strategies for people from ethnic minority backgrounds in an Australian health system context, we established that some existing strategies may be suitable but that adaptations were needed [13]. We therefore sought to develop an adapted patient engagement intervention using co-design with flexibility to meet the needs of a diverse patient population attending cancer services in New South Wales, Australia [14].

### The Making it Meaningful Intervention

Making it Meaningful (MiM) is a co-designed medication management tool that aims to increase patients’ and family members’ understanding of medication management to enable them to contact health care staff between appointments, and following hospital discharge in order to raise concerns about their medications. The MiM is an adaptation of an existing medication management tool used in New South Wales cancer services. The adapted form provides translated information about medication side effects, in plain language and using images in order to meet the needs of populations with low English language proficiency. During the co-design process to develop MiM, the participating service identified that people from Russian- and Mandarin-speaking populations have low English language proficiency patients and commonly attend the participating service. These 2 language groups are therefore targeted in this pilot study. Full details of the co-design and development process have been reported elsewhere [14]. The MiM is attached as Multimedia Appendix 1.

## Methods

### Aim

To determine whether MiM is feasible and acceptable for use with ethnic minority consumers in cancer services in Australia, this study will seek to address the following objectives: (1) to investigate how patients and staff engage with MiM; (2) to assess the feasibility and acceptability of MiM; (3) to investigate the suitability of trial recruitment, retention, and data collection materials and processes; (4) to explore barriers and enablers of implementing the MiM; (5) to gather evidence of the nature and frequency of medication management issues that lead to emergency department visits and readmissions to inform the sample size required for a full-scale trial; and (6) to determine whether a full-scale effectiveness trial is warranted.

### Design

This is a pilot, single-site, feasibility randomized controlled trial designed in concordance with SPIRIT guidelines (Multimedia Appendix 2).

### Setting

The setting is a metropolitan cancer service in New South Wales, Australia, providing surgery, oncology, radiotherapy, and palliative care in inpatient and outpatient settings.

### Sample

In total, 40 patients will be recruited to participate in this feasibility and acceptability study, 20 will take part in the control group using the current standard medication management tool and 20 in the intervention group using the MiM medication management tool.

### Eligibility Criteria

#### Inclusion Criteria

Inpatients and outpatients aged 18 years or older, who are receiving treatment or care at a participating cancer service, and identify their primary language as Russian or Mandarin will be eligible to take part.

#### Exclusion Criteria

Patients who are under 18 years old, who are not currently accessing a participating cancer service for their care, or do not identify Russian or Mandarin as a primary language are not eligible to participate.

### Recruitment

Potential participants who fulfill eligibility criteria will be provided written and verbal information about the study by their treating clinician or care coordinator in Russian or Mandarin, as appropriate. Potential participants will be asked by health service staff to indicate whether they wish for the research team to phone them to discuss the study, with no obligation to take part. A member of the research team will contact those who wish to learn more about the study via phone with support from an interpreter or multilingual worker if required. Consent will be audio-recorded with the participant’s permission and a translated consent form will be provided. This approach will make sure that accurate information is provided in an accessible form (ie, spoken in their first language and translated hard copy). Consent will further be confirmed verbally by the researcher prior to the commencement of telephone surveys.

### Randomization

Participants will be randomized to group 1 (intervention) or group 2 (comparison) during the recruitment process using a web-based randomization tool. Allocation will be stratified by language group.

**Figure 1:**
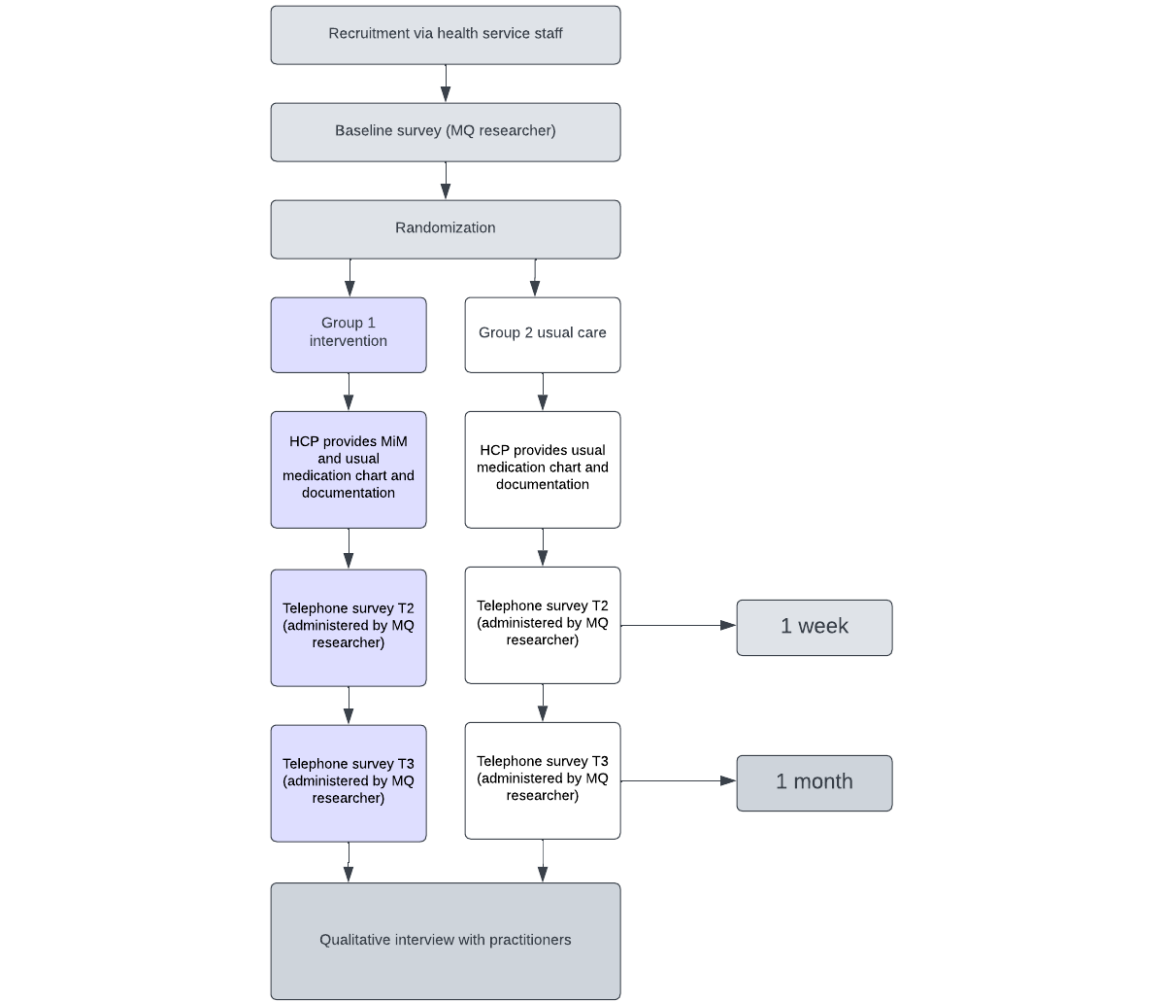
Flow diagram of data collection. HCP: health care practitioner; MiM: Making it Meaningful; MQ: Macquarie University; T2: time point 2, 1 week post intervention; T3: time point 3, 1 month post intervention.

Figure 1 provides a flow diagram describing the data collection process.

### Procedure

#### Intervention Group

Participants allocated to the intervention group will attend their usual consultation or appointment. The health practitioner (treating oncologist) will use MiM to facilitate communication at the appointment and provide a paper-translated copy of the MiM in Mandarin or Russian language as appropriate for the patient to take away from the appointment. Participants will take part in verbal phone surveys conducted by bilingual field-workers at 3 time points; before discharge or outpatient appointment, a week after discharge or outpatient appointment, and a month after discharge or outpatient appointment (Figure 1). These time points have been selected to provide opportunity for patients to use the MiM tool during and after they leave the appointment setting.

#### Control Group

Participants allocated to the control group will receive usual care from their health care practitioner, which includes communication about medications using the existing medication management tool available in the participating service. Participants will also complete a verbal phone survey at 3 time points (Figure 1).

### Ethics Approval

This protocol has been reviewed by the South Eastern Sydney Local Health District Human Research Ethics Committee and received approval for data collection. Appropriate ethics approvals have been obtained (HREC approval 2022/ETH01865). All participants will be required to provide consent prior to participation.

Informed consent will be gained from all participants prior to survey commencement, this will be confirmed verbally by bilingual workers in the participant’s preferred language at the time of the survey. For participating staff, written consent will be obtained prior to interview commencement.

All data will be stored on a secure, password-protected cloud-based system only accessible to the research team. All findings will be deidentified prior to sharing or reporting results beyond the research team.

Patient participants will receive an Aus $20 (is equal to US $12.81 [exchange rate accurate on 22 August 2023]) voucher to acknowledge their participation. This will be sent via post at the completion of each survey.

### Outcome Measures

#### Patients

An 11-item survey tool with closed and free-text items will be administered verbally over the phone (Multimedia Appendix 1). The survey comprises six components to capture (1) demographic information, (2) patient knowledge of medications and their side effects (n=4), (3) patient knowledge of how to contact the health service regarding medication concerns (n=3), (4) patient self-efficacy to contact the service about medication concerns (n=2), (5) patient engagement in medication management (n=3), and (6) patient perceptions of the safety of their care (n=2). Further, 2 systematic reviews of current measures of patient engagement demonstrated a lack of suitable existing scales for capturing consumer engagement in relation to discrete medication management activities relevant to the current project [15,16]. Drawing upon the key domains of patient engagement articulated in these reviews, we developed a set of 11 items to capture patient engagement in medication.

In total, 6 free-text items will be used in the final survey only to capture process information about the trial, including the feasibility of using MiM. This is to investigate the suitability of trial recruitment, retention, and data collection materials and processes (objective 3) and to explore barriers and enablers of implementing the MiM (objective 4). Researchers will seek to prevent pressure on patients to provide socially desirable responses by reminding the participants that researchers are independent of the health care team and this information is not being shared with their health care service or staff. The telephone survey will be administered by the research team supported by multilingual field-workers, at 3 time points (Figure 1). The researcher will record responses on Qualtrics (Qualtrics).

Researchers will contact participants via telephone and will attempt 2 follow-up calls. If participants are not available after 2 failed attempts to contact the patient the participating oncologist will be notified so that the patient contact details and willingness to participate can be confirmed. Phone surveys will be audio-recorded to facilitate the analysis of qualitative data.

#### Practitioners

Semistructured interviews with participating oncologists (health practitioners) will be conducted by a member of the research team at project completion, or when the practitioner finishes involvement with the project. Researchers will use a semistructured interview schedule to explore the feasibility of using the MiM in cancer services from a clinician perspective. This is to investigate the suitability of trial recruitment, retention, and data collection materials and processes (objective 3) and to explore barriers and enablers of implementing the MiM (objective 4).

#### Analytic Strategy

To address objectives 1-4, free-text data from the final patient survey and from the health practitioner interviews will be subject to thematic analysis. Further, 2 researchers will independently code data using Braun and Clarke’s 6-step approach to generate themes regarding patient and clinician experience using MiM, its feasibility and acceptability and factors influencing MiM implementation [17]. The clinician and patient data sets will be coded and analyzed separately, and then synthesized. Two researchers will (1) independently familiarize themselves with the data, (2) independently generate codes and then meet to confirm codes, (3) collaboratively construct themes, (4) review and revise potential themes in consultation with the research team, (5) define and name themes, and (6) finalize findings to address relevant objectives.

To inform objective 5, data about the nature and frequency of medication management issues will be obtained from survey data and recorded in an Excel spreadsheet. To address objective 6, data assessing the impact of MiM on knowledge about medication and self-efficacy for medication management will be compared between the control and intervention groups. Specifically, we will compare changes in self-efficacy for medication management, patient engagement, and patient perceived safety before and after the intervention, and between the intervention and control groups.

## Results

Appropriate ethical approval has been gained from the Human Research Ethics Committee and relevant governance body at the participating cancer service. Study enrollment has commenced with Mandarin-speaking participants; an oncologist and a bilingual field-worker. Survey collection will commence for users of Mandarin MiM participants who have been enrolled. Once data collection processes have been embedded with Mandarin-speaking patients, recruitment of participants from Russian-speaking backgrounds will commence.

## Discussion

### Principal Findings

This study seeks to determine whether MiM is feasible and acceptable for use with ethnic minority consumers in cancer services in Australia. The proposed study will test an adapted intervention that has been co-designed with people from ethnic minority backgrounds and health care staff to improve safety in the management of medication. By providing feasibility and process evaluation data about the MiM, trial materials and methods, this study will provide preliminary data about the implementation and impact of MiM on knowledge, behavior, and self-efficacy for people from Mandarin- and Russian-speaking backgrounds. We will gain insight into the use of the MiM tool but also of the research processes and outcome measures. Patient and practitioner views on the suitability and effectiveness of the MiM and commentary around useful features and suggested changes will provide the evidence base required to determine whether a full-scale effectiveness trial is warranted, and whether MiM, the study materials, or processes require adaptation to progress to a full trial. Current evidence highlights that many trials exclude or inhibit the participation of people from ethnic minority backgrounds, especially where there is low English language proficiency [18-20]. Determining through this research whether translated materials and support from multilingual field-workers is sufficient to support participation and data collection is critical to inform a full trial.

### Limitations

A key challenge for co-designed interventions is the difficulty often faced in seeking to implement interventions into practice [21]. By gathering evidence of the barriers and enablers to implementation of the MiM from patient and practitioner data collection, we seek to produce knowledge that supports services to facilitate the implementation process. While this study is limited to 2 language groups and in cancer services, there may be opportunities to explore the relevance and value of MiM for addressing medication safety in further population groups and health settings.

### Conclusions

This research is being conducted with a view to expanding the study to test whether the intervention is effective in improving patient outcomes in cancer care.

## Appendix

Multimedia Appendix 1 Making it Meaningful tool draft.

Multimedia Appendix 2 Spirit checklist.

## Abbreviations

MiM: Making it Meaningful

## Data Availability

Data and materials will be made available in publication of the pilot findings. The data sets generated or analyzed during this study will be available from the corresponding author on reasonable request.
