# Supplementary material for: Improving Medication Safety in Cancer Services for Ethnic Minority Consumers: Protocol for a Pilot Feasibility and Acceptability Study of a Co-Designed Consumer Engagement Intervention
Source: JMIR Res Protoc. 2023 Sep 18;12:e49902. doi: 10.2196/49902 (PMC10546273; doi:10.2196/49902)
Supplement: Multimedia Appendix 1 [file resprot_v12i1e49902_app1.pdf]

Name: **MRN:**

Health Care Practitioner:

SECTION 1 provides translated list of common side effects as a conversation tool and gives space for practitioner notes about specific treatment plans (in English)

**Possible side effects (Translated)**	
**English**	**Russian/Chinese**
Nausea/vomiting	
Headaches	
Itchy/Rash	
Confusion	
Reflux/heartburn	
Dizziness	
Diarrhoea	
Shortness of breath	
Swelling	
Chest pain	

NOTES:

PLEASE TURN OVER FOR PAGE 2

Medication used to treat cancer can sometimes cause serious problems. It is important to get medical help immediately if you become unwell.

If you have any questions about your treatment, appointments or plans.

If you are uncertain about medication side effects.

→

Call the care coordinator ……………………. ph. ………………..

The care coordinator is available on (add days and times)

or ……………………. ph.

If you experience:

- a temperature of 38ºC or higher
- chills, sweats, shivers
- difficulty breathing
- ongoing vomiting or diarrhoea
- pain, tingling or discomfort in your chest or arms
- you feel really unwell

→

Go to Emergency Department immediately . Call 000
