# Supplementary material for: Improving Medication Safety in Cancer Services for Ethnic Minority Consumers: Protocol for a Pilot Feasibility and Acceptability Study of a Co-Designed Consumer Engagement Intervention
Source: JMIR Res Protoc. 2023 Sep 18;12:e49902. doi: 10.2196/49902 (PMC10546273; doi:10.2196/49902)
Supplement: Multimedia Appendix 2 [file resprot_v12i1e49902_app2.pdf]

SUPPLEMENTARY FILE : **Standard Protocol Items: Recommendations for Interventional Trials (SPIRIT) checklist**

The SPIRIT checklist is copyrighted by the SPIRIT Group under the Creative Commons "Attribution-NonCommercial-NoDerivs 3.0 Unported" license. downloaded 13 June 2023 from https://www.spirit-statement.org/publications-downloads/

X indicates that this element was addressed,

NA indicates element not relevant to this pilot.

Completed Section/item ItemNo Description

Administrative information

X Title 1 Descriptive title identifying the study design, population, interventions, and, if applicable, trial acronym

X Trial registration 2a Trial identifier and registry name. If not yet registered, name of intended registry

2b All items from the World Health Organization Trial Registration Data Set

X Protocol version 3 Date and version identifier

X Funding4 Sources and types of financial, material, and other support

X Roles and responsibilities 5a Names, affiliations, and roles of protocol contributors

5b Name and contact information for the trial sponsor

5c Role of study sponsor and funders, if any, in study design; collection, management, analysis, and interpretation of data; writing of the report; and the decision to submit the report for publication, including whether they will have ultimate authority over any of these activities

5d Composition, roles, and responsibilities of the coordinating centre, steering committee, endpoint adjudication committee, data management team, and other individuals or groups overseeing the trial, if applicable (see Item 21a for data monitoring committee)

Introduction

X Background and rationale 6a Description of research question and justification for undertaking the trial, including summary of relevant studies (published and unpublished) examining benefits and harms for each intervention

6b Explanation for choice of comparators

X Objectives 7 Specific objectives or hypotheses

X Trial design 8 Description of trial design including type of trial (eg, parallel group, crossover, factorial, single group), allocation ratio, and framework (eg, superiority, equivalence, noninferiority, exploratory)

Methods: Participants, interventions, and outcomes

X Study setting 9 Description of study settings (eg, community clinic, academic hospital) and list of countries where data will be collected. Reference to where list of study sites can be obtained

X Eligibility criteria 10 Inclusion and exclusion criteria for participants. If applicable, eligibility criteria for study centres and individuals who will perform the interventions (eg, surgeons, psychotherapists)

X Interventions 11a Interventions for each group with sufficient detail to allow replication, including how and when they will be administered

11b Criteria for discontinuing or modifying allocated interventions for a given trial participant (eg, drug dose change in response to harms, participant request, or improving/worsening disease)

11c Strategies to improve adherence to intervention protocols, and any procedures for monitoring adherence (eg, drug tablet return, laboratory tests)

11d Relevant concomitant care and interventions that are permitted or prohibited during the trial

X Outcomes 12 Primary, secondary, and other outcomes, including the specific measurement variable (eg, systolic blood pressure), analysis metric (eg, change from baseline, final value, time to event), method of aggregation (eg, median, proportion), and time point for each outcome. Explanation of the clinical relevance of chosen efficacy and harm outcomes is strongly recommended

X Participant timeline 13 Time schedule of enrolment, interventions (including any run-ins and washouts), assessments, and visits for participants. A schematic diagram is highly recommended (see Figure)

X Sample size 14 Estimated number of participants needed to achieve study objectives and how it was determined, including clinical and statistical assumptions supporting any sample size calculations

X Recruitment 15 Strategies for achieving adequate participant enrolment to reach target sample size

Methods: Assignment of interventions (for controlled trials)

X Allocation:

Sequence generation 16a Method of generating the allocation sequence (eg, computer-generated random numbers), and list of any factors for stratification. To reduce predictability of a random sequence, details of any planned restriction (eg, blocking) should be

provided in a separate document that is unavailable to those who enrol participants or assign interventions

NA	Allocation concealment mechanism	16b	Mechanism of implementing the allocation sequence (eg, central telephone; sequentially numbered, opaque, sealed envelopes), describing any steps to conceal the sequence until interventions are assigned

X	Implementation16c	Who will generate the allocation sequence, who will enrol participants, and who will assign participants to interventions

X	Blinding (masking)	17a	Who will be blinded after assignment to interventions (eg, trial participants, care providers, outcome assessors, data analysts), and how

NA		17b	If blinded, circumstances under which unblinding is permissible, and procedure for revealing a participant's allocated intervention during the trial

Methods: Data collection, management, and analysis

X	Data collection methods	18a	Plans for assessment and collection of outcome, baseline, and other trial data, including any related processes to promote data quality (eg, duplicate measurements, training of assessors) and a description of study instruments (eg, questionnaires, laboratory tests) along with their reliability and validity, if known. Reference to where data collection forms can be found, if not in the protocol

18b	Plans to promote participant retention and complete follow-up, including list of any outcome data to be collected for participants who discontinue or deviate from intervention protocols

X	Data management	19	Plans for data entry, coding, security, and storage, including any related processes to promote data quality (eg, double data entry; range checks for data values). Reference to where details of data management procedures can be found, if not in the protocol

X	Statistical methods	20a	Statistical methods for analysing primary and secondary outcomes. Reference to where other details of the statistical analysis plan can be found, if not in the protocol

20b	Methods for any additional analyses (eg, subgroup and adjusted analyses)

20c	Definition of analysis population relating to protocol non-adherence (eg, as randomised analysis), and any statistical methods to handle missing data (eg, multiple imputation)

Methods: Monitoring

NA	Data monitoring	21a	Composition of data monitoring committee (DMC); summary of its role and reporting structure; statement of whether it is independent from the sponsor and competing interests; and reference to where further details about its charter can be found, if not in the protocol. Alternatively, an explanation of why a DMC is not needed

21b	Description of any interim analyses and stopping guidelines, including who will have access to these interim results and make the final decision to terminate the trial

X	Harms	22	Plans for collecting, assessing, reporting, and managing solicited and spontaneously reported adverse events and other unintended effects of trial interventions or trial conduct

X Auditing 23 Frequency and procedures for auditing trial conduct, if any, and whether the process will be independent from investigators and the sponsor

Ethics and dissemination

X Research ethics approval 24 Plans for seeking research ethics committee/institutional review board (REC/IRB) approval

NA Protocol amendments 25 Plans for communicating important protocol modifications (eg, changes to eligibility criteria, outcomes, analyses) to relevant parties (eg, investigators, REC/IRBs, trial participants, trial registries, journals, regulators)

X Consent or assent 26a Who will obtain informed consent or assent from potential trial participants or authorised surrogates, and how (see Item 32)

26b Additional consent provisions for collection and use of participant data and biological specimens in ancillary studies, if applicable

X Confidentiality 27 How personal information about potential and enrolled participants will be collected, shared, and maintained in order to protect confidentiality before, during, and after the trial

x Declaration of interests 28 Financial and other competing interests for principal investigators for the overall trial and each study site

x Access to data 29 Statement of who will have access to the final trial dataset, and disclosure of contractual agreements that limit such access for investigators

NA Ancillary and post-trial care 30 Provisions, if any, for ancillary and post-trial care, and for compensation to those who suffer harm from trial participation

x Dissemination policy 31a Plans for investigators and sponsor to communicate trial results to participants, healthcare professionals, the public, and other relevant groups (eg, via publication, reporting in results databases, or other data sharing arrangements), including any publication restrictions

31b Authorship eligibility guidelines and any intended use of professional writers

31c Plans, if any, for granting public access to the full protocol, participant-level dataset, and statistical code

Appendices

Available on request Informed consent materials 32 Model consent form and other related documentation given to participants and authorised surrogates

NA NA Biological specimens 33 Plans for collection, laboratory evaluation, and storage of biological specimens for genetic or molecular analysis in the current trial and for future use in ancillary studies, if applicable

The SPIRIT checklist is copyrighted by the SPIRIT Group under the Creative Commons “Attribution-NonCommercial-NoDerivs 3.0 Unported” license.
